# Effects of Deoxynivalenol and Zearalenone on the Histology and Ultrastructure of Pig Liver

**DOI:** 10.3390/toxins12070463

**Published:** 2020-07-20

**Authors:** Natalia Skiepko, Barbara Przybylska-Gornowicz, Magdalena Gajęcka, Maciej Gajęcki, Bogdan Lewczuk

**Affiliations:** 1 Department of Histology and Embryology, Faculty of Veterinary Medicine, University of Warmia and Mazury in Olsztyn, Oczapowskiego 13, 10-719 Olsztyn, Poland; natalia.skiepko@uwm.edu.pl (N.S.); przybyl@uwm.edu.pl (B.P.-G.); 2 Department of Veterinary Prevention and Feed Hygiene, Faculty of Veterinary Medicine, University of Warmia and Mazury in Olsztyn, Oczapowskiego 13, 10-719 Olsztyn, Poland; mgaja@uwm.edu.pl (M.G.); gajecki@uwm.edu.pl (M.G.)

**Keywords:** zearalenone, deoxynivalenol, mycotoxins, histology, ultrastructure, pig, hepatocyte, liver

## Abstract

The purpose of this study was to determine the effects of single and combined administrations of deoxynivalenol (DON) and zearalenone (ZEN) on the histology and ultrastructure of pig liver. The study was performed on immature gilts, which were divided into four equal groups. Animals in the experimental groups received DON at a dose of 12 μg/kg body weight (BW) per day, ZEN at 40 μg/kg BW per day, or a mixture of DON (12 μg/kg BW per day) and ZEN (40 μg/kg BW). The control group received vehicle. The animals were killed after 1, 3, and 6 weeks of experiment. Treatment with mycotoxins resulted in several changes in liver histology and ultrastructure, including: (1) an increase in the thickness of the perilobular connective tissue and its penetration to the lobules in gilts receiving DON and DON + ZEN; (2) an increase in the total microscopic liver score (histology activity index (HAI)) in pigs receiving DON and DON + ZEN; (3) dilatation of hepatic sinusoids in pigs receiving ZEN, DON and DON + ZEN; (4) temporary changes in glycogen content in all experimental groups; (5) an increase in iron accumulation in the hepatocytes of gilts treated with ZEN and DON + ZEN; (6) changes in endoplasmic reticulum organization in the hepatocytes of pigs receiving toxins; (7) changes in morphology of Browicz–Kupffer cells after treatment with ZEN, DON, and DON + ZEN. The results show that low doses of mycotoxins used in the present study, even when applied for a short period, affected liver morphology.

## 1. Introduction

Mycotoxins, secondary fungal metabolites, are frequent contaminants of cereals and cereal products. Delivered via these plants and products, they pose a serious health threat to humans and animals [1,2,3]. Whereas mycotoxins are sometimes regarded as stressors [4,5], they have a target-specific mode-of-action and so are true toxins rather than stressors per se [6]. The most common and important mycotoxins in Europe, produced by fungi of the *Fusarium* family, are deoxynivalenol (DON), and zearalenone (ZEN). Pigs are particularly sensitive to DON [7], but they also show high sensitivity to ZEN [8].

In pigs, high doses of DON cause reduced appetite, complete anorexia, vomiting [9], and reproductive disorders [10]. Absorption of DON is rapid, and the toxin reaches the peak plasma concentrations within 30 min of oral administration [11]. The majority of ingested DON is absorbed in the proximal part of the small intestine [12]. In the liver, DON is metabolized into de-epoxy-DON [13]. An evidence showed that chronic ingestion of DON at low doses, which is clinically asymptomatic, alters the mucosal epithelial cells and villi of the small intestine [14,15,16,17] and affects the defense mechanisms of the large intestine [18].

ZEN and its metabolites are agonists of estrogen receptors, and they compete with endogenous hormones for the binding sites of estrogen receptors [2,19]. Treatment with ZEN leads to precocious puberty, reproductive disorders, and hyperestrogenism [20]. According to previous studies, domestic pigs are particularly sensitive to the estrogenic effect of ZEN owing to the very rapid and large (approximately 80–85%) absorption of the toxin in their digestive system [21]. In the liver, ZEN is metabolized into α- and β-zearalenol, which are considered more toxic than ZEN. The ratio of these two metabolites to each other is species-specific. Most studies showed that α-zearalenol predominates in pigs [13,21,22,23,24,25]. This form is more active than β-zearalenol, which provides another explanation for the high sensitivity of pigs to ZEN content in feed [21,24]. ZEN at low doses has deleterious effects on the morphology of the small intestine [16,17] and affects the defense mechanisms of the liver [26] and the large intestine [18].

During exposure to environmental mycotoxins, animals often encounter mixtures of toxins, rather than single toxins. Therefore, the toxicity of mycotoxins needs to be addressed in the context of their mixtures to assess their health risk [27,28,29]. However, studies on the effects of mycotoxin combinations are relatively rare, and their results are very ambiguous [27,28,30,31].

The liver and gastrointestinal parts of the digestive system are major sites of mycotoxin metabolism [32]. However, there is little molecular, metabolic, and histological research on the effects of mycotoxins on the liver. In addition, the outcomes of these studies are not conclusive [15,28]. In pigs, some histological changes have been observed in the liver as a result of the individual or combined effects of DON and ZEN [15,33]. Contrastingly, Renner and coauthors [34] showed that chronic dietary DON exposure (4 weeks, 4.59 µg/kg BW) did not affect the histology of pig liver.

The purpose of this study was to determine the effects of single and combined administrations of DON (12 μg/kg BW) and ZEN (40 μg/kg BW) to pigs for 1, 3, or 6 weeks on the histology and ultrastructure of the liver. The selection of the doses used has been widely discussed in our previously published papers [16,17,18].

## 2. Results and Discussion

### 2.1. Light Microscopy Study

#### 2.1.1. Architecture of the Liver

**Figure 1:**
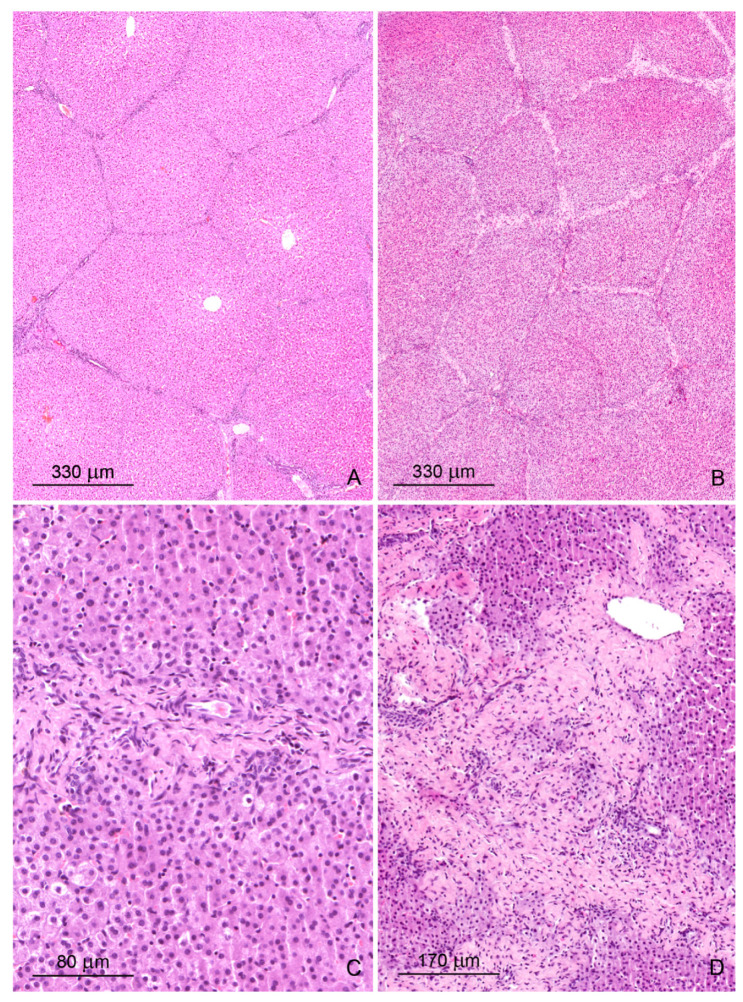
(**A**,**B**) Architecture of the liver in a control pig, 6th week of experiment (**A**) and in a pig receiving deoxynivalenol (DON) for 6 weeks (**B**). Note the thickening of the interlobular septa. (**C**) A strip of connective tissue in the liver lobule. A pig was treated with DON for 3 weeks. (**D**) Focal fibrosis inside the liver lobule of a pig treated with DON for 6 weeks. Note that the central vein is surrounded by connective tissue. Figures (**A**–**D**) show hematoxylin and eosin stained sections.

**Figure 2:**
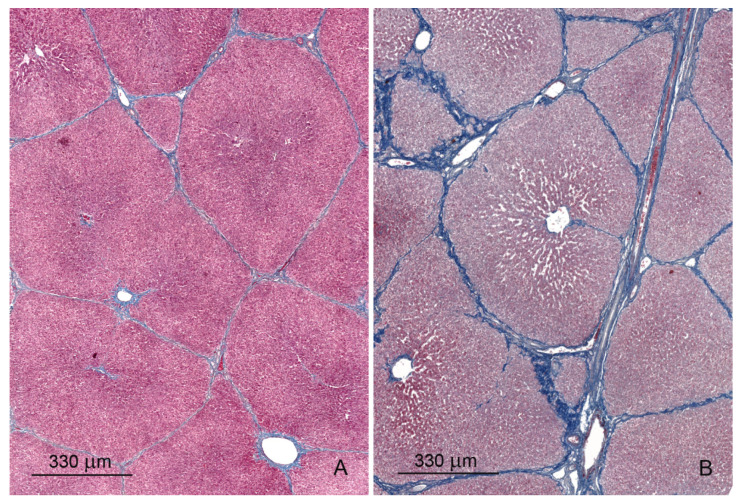
(**A**,**B**) Trichrome staining of the liver in a control pig, 6th week of experiment (**A**) and in a pig receiving DON for 6 weeks (**B**). Pay attention to the thickness of the interlobular septa and the content of collagen fibers in them.

The livers of control pigs had the typical structural characteristic for this species (Figure 1A and Figure 2A). The hepatic lobules were neatly outlined by an envelope of fibrous connective tissue, which interconnected the portal areas. Within each lobule, hepatocytes were arranged in linear cords radiating from the central vein and separated by sinusoids, which had a uniform diameter over their entire length. The limiting plate of hepatocytes bordered the lobule interior from the connective tissue.

Prominent qualitative differences in the liver architecture were observed between the control group and the DON and DON + ZEN groups. The perilobular connective tissue widened and contained greater amounts of collagen fibers starting from the first week of treatment (Figure 1B and Figure 2B, Table 1). In addition, connective tissue penetrated into the lobule and caused disruption of the limiting plate (Figure 1C). After 6 weeks of treatment, the presence of small scars was pronounced (Figure 1D). The disorganization of hepatic cords was observed in the livers of pigs fed diets contaminated with DON and DON + ZEN for 3 and 6 weeks.

**Figure 3:**
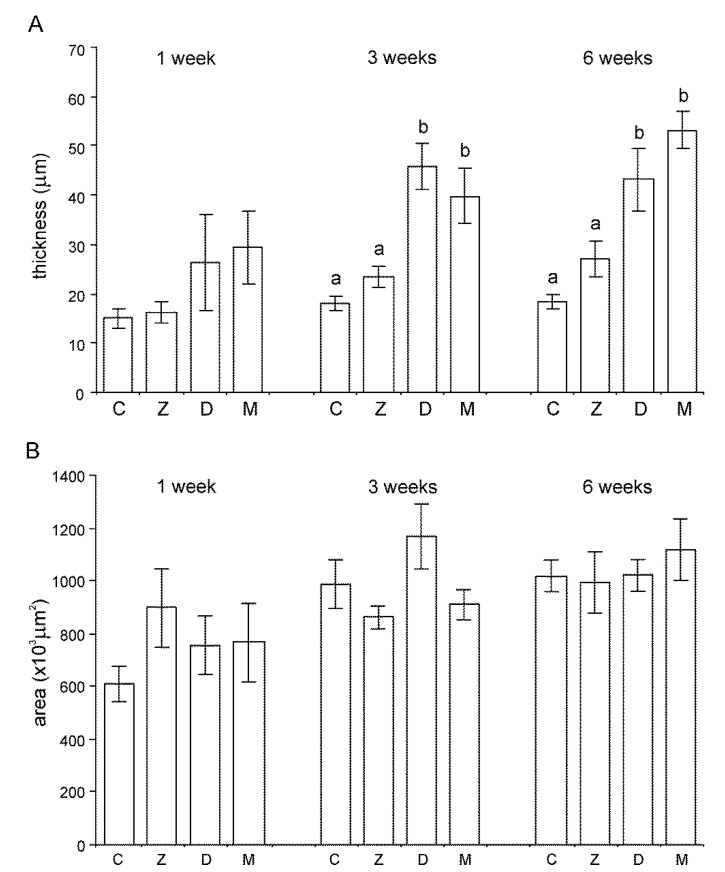
(**A**) Thickness of the connective tissue septa. (**B**) Cross-sectional area of the liver lobules. C—control group, Z—group treated with zearalenone (ZEN), D—group treated with deoxynivalenol (DON), and M—group treated with DON and ZEN. The values are presented as mean ± standard deviation. Bars labeled with different small lower-case letters differ significantly at *p* ≤ 0.05.

The administration of DON and DON + ZEN significantly increased the thickness of perilobular connective tissue in the liver after 3 and 6 weeks of treatment. No significant differences were observed in the thickness of septa after ZEN treatment (Figure 3A). The cross-sectional area of the lobules did not differ between the control group and the groups treated with mycotoxins (Figure 3B).

The data show that DON and DON + ZEN, but not ZEN, affected the qualitative and quantitative characteristics of the liver architecture. The administration of DON and DON + ZEN resulted in an excessive accumulation of extracellular matrix, including collagen, in perilobular connective tissue, starting from the first week of treatment. Moreover, treatment with DON + ZEN for 3 and 6 weeks caused a progressive accumulation of extracellular matrix in the liver parenchyma. We interpreted the intra-lobular presence of mild scars as early fibrosis. The process of liver fibrosis is most often a consequence of chronic diseases, but it can also be caused by many factors that induce acute damage [35,36]. In experimental studies, administration of xenobiotics induced parenchymal fibrosis similar to cirrhosis in rodent liver [36]. The intensity of the fibrosis process caused by external factors shows large differences [37]. Previous studies have shown that mycotoxins affect the histology of pig liver [15,34,38]; however, the authors did not report the process of liver fibrosis.

Disorganization of hepatic cords as one of the main lesions in piglets after chronic exposure to DON was observed by Gerez et al. [15]. In our study, similar changes were found after 6 weeks of DON and DON + ZEN ingestion.

#### 2.1.2. Microscopic Liver Scoring

**Figure 4:**
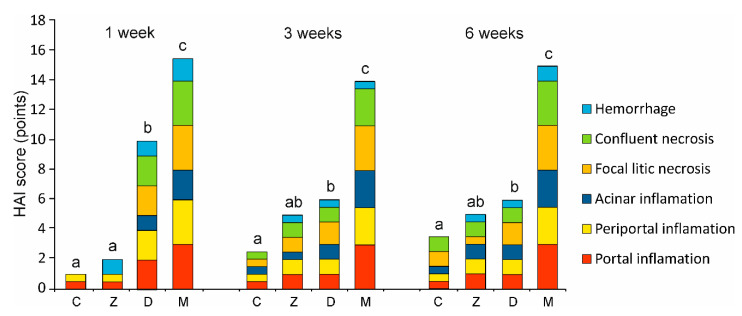
Histopathological score of the examined livers according to the histology activity index (HAI) modified by Stanek et al. [38]. Six histopathological parameters were scored in hematoxylin and eosin (HE)-stained tissues (see Figure S1 in Supplementary material), and the cumulative HAI score was calculated for each experimental group. The mean value of the cumulative HAI scores in each group is represented by the total height of the bar. C—control group, Z—group treated with zearalenone (ZEN), D—group treated with deoxynivalenol (DON), and M—group treated with DON and ZEN. The values of the cumulative HAI score labeled with different lower-case letters above the bars differ significantly at *p* ≤ 0.05.

The analysis using modified microscopic liver scoring (histology activity index (HAI)) comprised six histological criteria. Exemplary microphotographs of changes included in the individual criteria are presented in Figure S1 (Supplementary Material). The cumulative score and the contribution of each criterion are presented in Figure 4. The largest histopathological lesions of the liver were observed in pigs treated with DON for 1 week or DON + ZEN for 1, 3, 6 weeks. The total HAI score for DON resulted mainly from increased portal inflammation and focal lytic necrosis, whereas that for DON + ZEN resulted from increased portal and periportal inflammation, confluent necrosis, and especially focal lytic necrosis, compared with control pigs. The total HAI scores in pigs receiving DON and DON + ZEN for 1, 3, and 6 weeks were significantly higher than in that in the control group.

The foci of hepatocyte necrosis with lymphocytic infiltrates, portal, periportal, and acinar inflammation occur in the liver as a result of drug usage, intoxication, or viral and bacterial infections [39]. The occurrence of necrosis is also associated with the activation of immune mechanisms in response to the adverse effects of toxic substances [36,40]. These changes are generally associated with pathological symptoms; however, they also occur in seemingly healthy animals, and, in this case, they are considered as preclinical conditions [41].

The results of our HAI analysis showed that the liver, being the first metabolic station, is affected to varying degrees by the administered toxins. Treatment with ZEN had no significant effect on liver histopathology. However, DON and DON + ZEN significantly affected pig liver regardless of the administration period of toxins. The highest intensity of necrosis foci was observed in the livers of animals receiving both mycotoxins. The results obtained may be interpreted as an effect of the toxic effects of DON and the synergistic toxic effect of the combined toxins. In addition, they may be caused by the stimulation of inflammatory processes in response to the administered mycotoxins. This is due to the pro-inflammatory actions of DON and ZEN [42].

The results of previous studies on the effect of mycotoxins on the histology of the liver parenchyma are diverse. Histological changes, including the disorganization of hepatic cords, the cytoplasmic vacuolization of hepatocytes, megalocytosis, and focal necrosis, were reported in pigs subjected to 28 days of diet contaminated with DON (3 mg/kg) or DON (3 mg/kg) + NIV (1,5 mg/kg) + ZEN (1,5 mg/kg) [15]. Acute exposure to DON at a dose of 1 mg/kg BW for 6 and 24 h led to apoptosis of hepatocytes [33]. On the other hand, exposure to DON at a concentration of 4.59 mg/kg feed for 28 days [34] or 3.1 mg/kg feed for 37 days did not affect liver HAI [38].

Megalocytosis and cytoplasmic vacuolization were the main histological lesions reported in piglets subjected to chronic exposure to DON [15]. Magalocytosis potentially indicates irreversible hepatocyte injury [43]. However, in our study, this phenomenon concerned only a few hepatocytes, and there were no differences in their presence between control and experimental pigs.

#### 2.1.3. Hepatic Sinusoids

The livers of pigs from all experimental groups were characterized by significant dilatation of the hepatic sinusoids in Zone III (drainage) of acinus compared with those of control animals (Table 2; Supplementary material, Figure S2). The largest sinusoidal dilatation was observed in pigs receiving DON + ZEN. In these animals, significant sinusoidal dilatation occurred after 1, 3, and 6 weeks of mycotoxin administration. In the groups treated with ZEN alone and DON alone, sinusoidal dilatation decreased after 3 and 6 weeks (Table 2).

Under physiological conditions, the liver sinusoids are characterized by a constant, uniform diameter. Sinusoidal dilatation in Zone I has been observed in pregnancy, long-term steroid administration, and hepatomegaly [44]. This phenomenon also occurs after exposure to vinyl chloride and arsenic [40], and may also be the result of the administration of certain chemotherapy drugs [45]. Sinusoidal dilatation in Zone III was observed in the case of local disorders of blood flow in the portal veins or hepatic veins [40]. In our study, pronounced sinusoidal dilatation in Zone III was observed as a result of DON and ZEN administration, which suggested a disruptive effect of mycotoxins on blood flow in the liver. However, the histological findings did not provide any suggestions about the possible cause of this disturbance.

#### 2.1.4. Glycogen Storage

**Figure 5:**
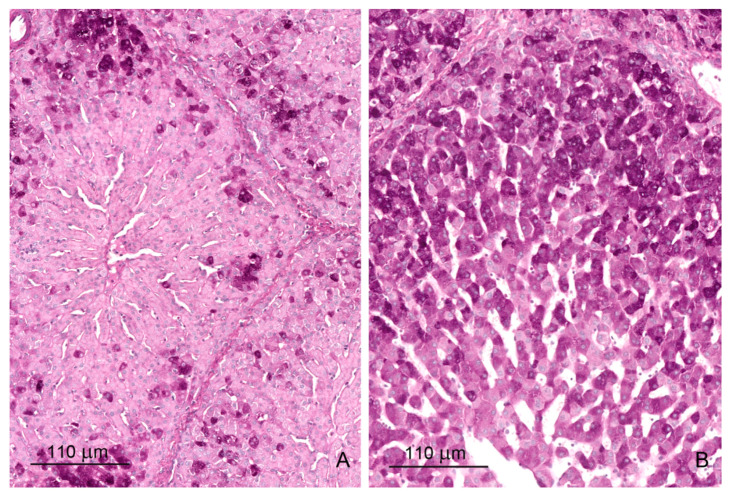
(**A**,**B**) Distribution of glycogen in hepatocytes in a control pig (**A**) and a pig treated with ZEN + DON (**B**). The first week of experiment. Periodic acid-Schiff reaction. Note the increase in the amount of glycogen in a pig treated with mycotoxins.

We used periodic acid-Schiff (PAS) staining to evaluate changes in glycogen content. An increase in glycogen deposits was observed in the groups treated with ZEN, DON and DON + ZEN compared with those in the control group after the first week of experiment (Figure 5, Table 3). The staining was particularly intense in Zone I. After 3 and 6 weeks of treatment, there were no differences in glycogen content between the control and experimental groups.

The liver is a major site of glycogen accumulation, and the main role of glycogen in the liver is to store glucose for release during fasting. The amount of stored glycogen depends on nutritional and living factors and shows large individual fluctuations [36,40]. In previous studies, no effects of mycotoxins on glycogen storage were observed in pig liver [7]. The results of our current studies showed that the effect of DON and ZEN on liver glycogen was temporary.

#### 2.1.5. Iron Deposits

**Figure 6:**
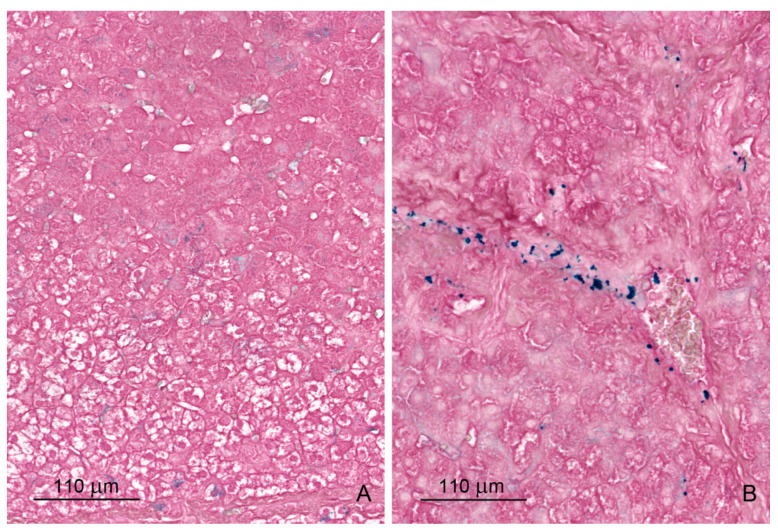
(**A**,**B**) Iron deposits in the liver of pigs receiving ZEN + DON for six weeks. (**A**) Iron deposits in hepatocytes in the form of fine granules. (**B**) Iron deposits with variable sizes in perilobular connective tissue. Prussian blue staining.

Prussian blue (PB) staining was used for the visualization of iron deposits. In control pigs, small amounts of iron deposits were observed in hepatocytes and Browicz–Kupffer cells in the form of very fine (pollen) granules. Iron-loaded cells are either isolated or grouped together without any lobular systematization. In connective tissues, positive staining for iron corresponds to deposition within fibrocytes or macrophages. In experimental animals, increased amounts of iron deposits were observed both in the liver lobules and perilobular connective tissues, especially after 6 weeks of ZEN and DON + ZEN administration (Figure 6, Table 4).

Physiologically, iron deposits are present within hepatocytes as fine granules at the biliary pole of cells and are distributed throughout the lobule according to a decreasing gradient from periportal to centrilobular areas. Iron excess is associated with chronic liver diseases of various causes [46,47,48]. It is known that in states of persistent iron overload, the liver could be seriously affected [49], mainly because of the extremely reactive hydroxyl radical formed during Fe^2+^ metabolism (by Fenton reaction) in lysosomes [50].

Previous studies on the effect of mycotoxins on pig liver did not consider the effect of ZEN on iron storage [7]. Based on the results obtained in our study, it can be stated that ZEN induced duration-dependent iron accumulation in hepatocytes, Browicz–Kupffer cells, and connective tissue cells. This indicated that disruption of iron metabolism in the liver worsened along with the duration of ZEN administration. One possible mechanism is the effect of ZEN on hepcidin, a liver-derived peptide hormone, which is a master regulator of iron metabolism [50]. Our results show a similar effect of food contaminated with DON + ZEN on iron accumulation in the liver, as in the case of ZEN, which suggested that DON had no effect on iron accumulation.

### 2.2. Ultrastructural Study

#### 2.2.1. Hepatocytes

**Figure 7:**
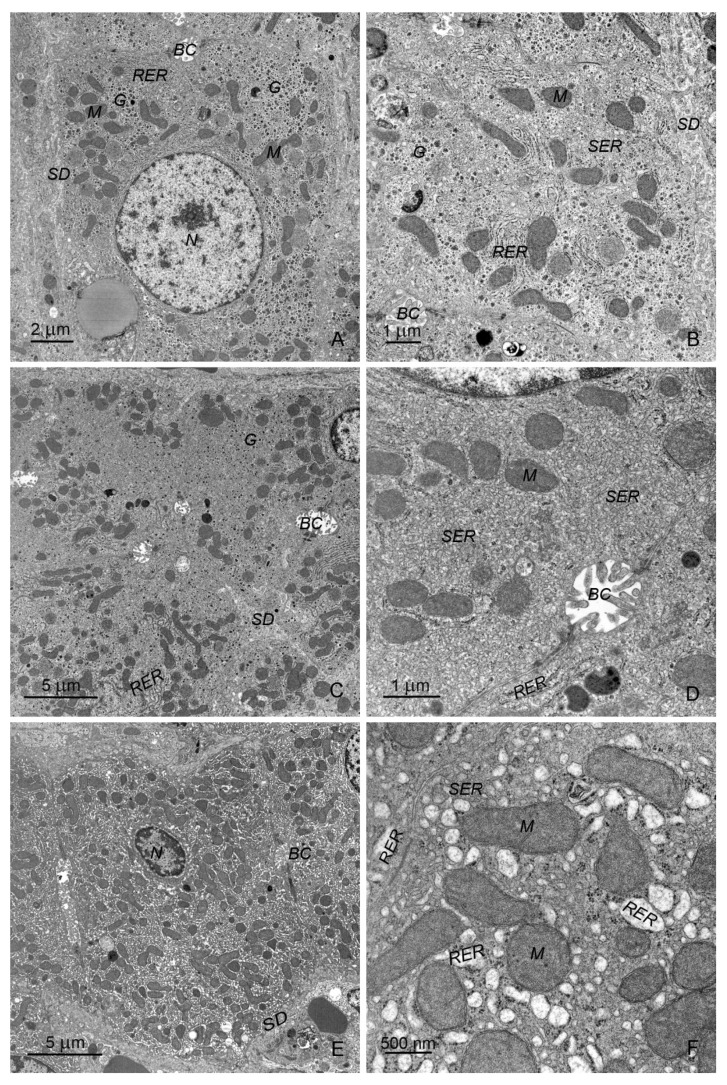
(**A**–**F**) Ultrastructure of hepatocytes in control pigs, 6th week of the experiment (**A**,**B**), pigs treated with ZEN for 6 weeks (**C**,**D**) and pigs treated with DON for 6 weeks (**E**,**F**). Note the specific organization of endoplasmic reticulum with the predominance of smooth reticulum over rough reticulum in pigs treated with ZEN. In DON-treated animals, both types of endoplasmic reticulum were formed by abundant short, dilated cisterns and vesicles. SD, space of Disse; BC, bile canaliculus; N, nucleus; M, mitochondria; RER, rough endoplasmic reticulum; SER, smooth endoplasmic reticulum; G, glycogen particles.

The hepatocytes of control pigs (Figure 7A) with polygonal or rectangular cross sections were arranged in regular rows. Their nuclei were distinctly round and centrally located, with a predominance of euchromatin. Hepatocytes had abundant amounts of both rough and smooth endoplasmic reticulum (ER). Medium-length cisterns of rough ER were located close to the nucleus and around the mitochondria (Figure 7B). The smooth ER was in the form of branched tubules and vesicles (Figure 7B). Mitochondria with numerous cristae and an electron-dense matrix were distributed in the cytoplasm in clusters or individually (Figure 7A). Lysosomes were localized mainly in the bile pole of hepatocytes. The content of glycogen deposits varied significantly between individual hepatocytes. After the first week of experiment, glycogen deposits were rather sparse; however, they were more numerous after three and six weeks. Lipid droplets were few and randomly distributed in the cytoplasm of hepatocytes. The vascular domain surface of hepatocytes was covered by numerous microvilli.

The differences in hepatocyte ultrastructure in experimental pigs compared with control animals were evident after 1, 3, and 6 weeks of treatment. The hepatocytes of ZEN-treated animals were characterized by a specific ER (Figure 7C,D). The smooth ER consisted of a network of closely located very narrow tubules and small vesicles, and occupied most of the cross sections of the hepatocytes. The poorly developed rough ER created several clusters of cisterns arranged in parallel. The mitochondria were located near these cisterns. Changes in the ER were observed from the first week of experiment; however, the intensity increased markedly after 3 and 6 weeks of treatment with ZEN. The ultrastructure of the mitochondria and lysosomes in ZEN-treated pigs did not differ significantly from that in the control animals. However, occasionally, some damaged mitochondria were observed. The amount of glycogen deposits in hepatocytes was usually low, except in the samples taken after the first week of the experiment. Some necrotic hepatocytes were noted, especially after 6 weeks of treatment.

In hepatocytes of DON-treated pigs, both types of ER were formed by abundant short, dilated cisterns and vesicles (Figure 7E,F), starting from the first week of the experiment. Both types of ER were quite evenly distributed in the cytoplasm of hepatocytes. In some cells, very dilated vacuoles of rough ER filled with protein micelles were observed. Large autophagosomes were present in the cytoplasm of numerous hepatocytes. Necrotic cells were occasionally observed.

The ultrastructure of hepatocytes in DON + ZEN-treated pigs did not differ from that in DON-treated pigs, except that necrotic hepatocytes were more frequently observed.

Changes in hepatocyte ultrastructure accompany numerous subclinical and clinical intoxications, both acute and chronic. The nature of these changes depends on the chemical properties of the toxin, dose, and duration of exposure. The most common changes are smooth and rough ER swelling, damage of mitochondria, and modification in the amount and distribution of lipid drops and glycogen deposits [51]. To date, data on the effect of mycotoxins on the ultrastructure of hepatocytes in pigs are scarce [7].

Our results show that the mycotoxins used in the experiment affected the ultrastructure of hepatocytes, especially the ER. ZEN administration resulted in conspicuous smooth ER proliferation and rough ER marginalization. Smooth ER is involved in the metabolism of various chemicals, and cells exposed to these chemicals showed hypertrophy of the smooth ER as an adaptive response. For this reason, smooth ER hypertrophy is considered a sensitive toxicological parameter [52].

The administration of DON and DON + ZEN resulted in the dilatation of ER cisterns and no clear difference between the two types of reticulum. The dilatation of ER cisternae, which indicates a loss of ER homeostasis, is known as ER stress [53]. ER stress is associated with liver injury and fibrosis. The hepatic factors that regulate ER stress remain unknown [54]. The fact that DON and DON + ZEN induced ER dilatation suggested that chronic ingestion of low doses of these mycotoxins caused injury in pig hepatocytes.

#### 2.2.2. Sinusoids and Perivascular Species

Hepatic sinusoids were formed by flat endothelial cells with prominent fenestrations, usually lacking the basal lamina. Browicz–Kupffer cells with electron-lucent cytoplasm and numerous granules with variable appearance were located inside the sinusoids. Ito cells situated outside the vessels were numerous and usually contained one lipid droplet of moderate size. Pit cells situated inside the vessels were sporadically observed.

**Figure 8:**
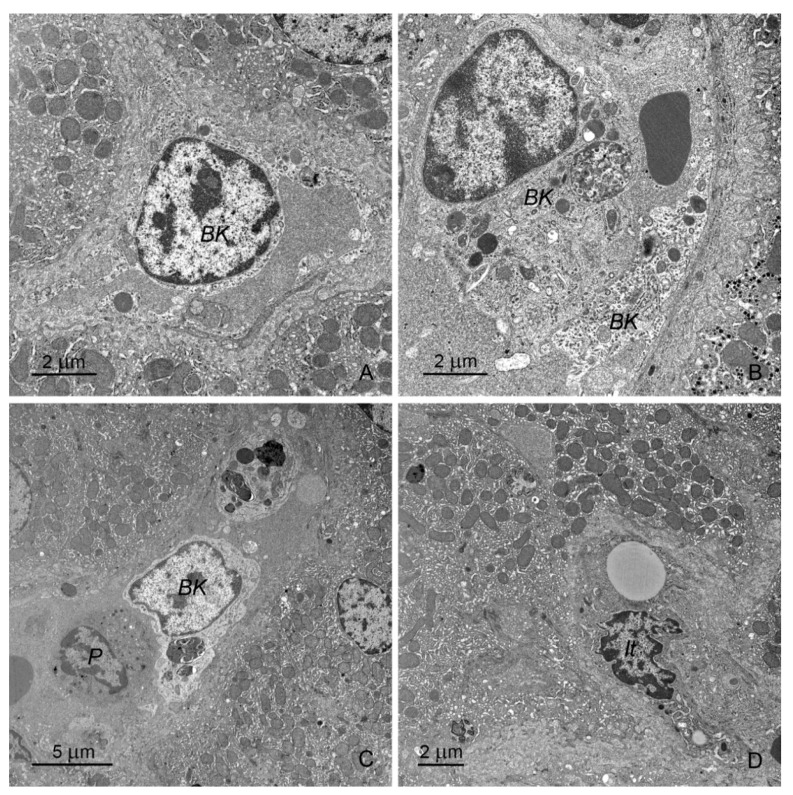
(**A**,**B**) Browicz–Kupffer cells (BK) in the sinusoid of pigs treated with DON for 3 weeks (**A**) and 6 weeks (**B**). Note the presence of numerous processes in Figure (**A**) and lysosomes and rest bodies in Figure (**B**,**C**). BK and pit (P) cells in the sinusoid of a pig treated with DON + ZEN for 6 weeks. (**D**) Ito cell (It) in the perivascular space of pigs treated with DON + ZEN for 6 weeks. Note the cisterns of the rough endoplasmic reticulum.

There were no differences in the ultrastructure of endothelial cells between control pigs and animals receiving mycotoxins. Browicz–Kupffer cells appeared to be more numerous in pigs receiving ZEN for 1 week as well as in pigs treated with DON and DON + ZEN for 1, 3, or 6 weeks than those in the control animals (Figure 8A–C). In these animals, Browicz–Kupffer cells showed prominent cell processes and occupied a large part of the vessel lumen. Their cytoplasm contained numerous phagosomes and rest bodies. Pit cells were more frequently observed in pigs treated with DON and DON + ZEN for 3 and 6 weeks than in the control animals (Figure 8C). In pigs treated with DON and DON + ZEN for 1, 3, or 6 weeks Ito cells were characterized by the presence of prominent cisterns of the rough ER (Figure 8D).

**Figure 9:**
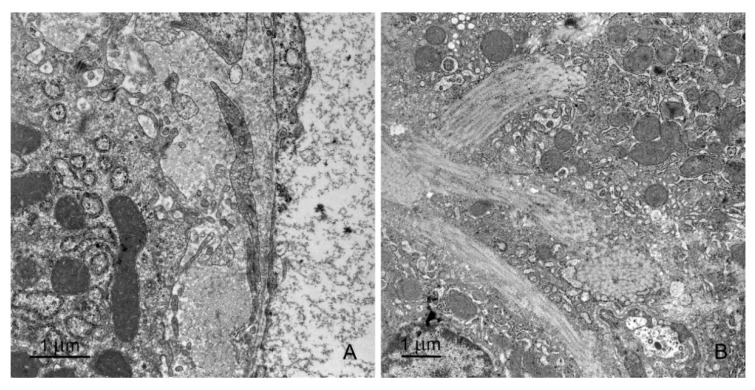
(**A**) Accumulation of collagen fiber in the space of Disse in a pig receiving DON for 6 weeks. (**B**) Penetration of collagen fibers between hepatocytes in a pig treated with DON + ZEN for 6 weeks.

The focal accumulation of collagen fibers was observed in the perivascular spaces of pigs receiving DON and DON + ZEN for 3 and 6 weeks (Figure 9A). Their number increased with the duration of treatment. The penetration of collagen fibers between hepatocytes and foci of fibrosis was noted in pigs receiving DON + ZEN for 3 and 6 weeks (Figure 9B).

Our data shows, for the first time, that the administration of mycotoxins, especially DON, induced changes in the population of Browicz–Kupffer cells, which suggested their activation. These cells play an important role in the clearance of toxins from the portal blood [55]. Several cytokines, chemokines, and reactive nitrogen and oxygen species are released by activated Browicz–Kupffer cells, allowing these cells to modulate microvascular responses and the functions of hepatocytes and Ito cells [55,56]. Browicz–Kupffer cells show large plasticity, adopting changes in local metabolic and immune environment. They can play a protective role through their tolerogenic phenotype in toxin-induced liver injury, but can also shift to a pathologically activated state and contribute to liver inflammation. It could be considered that the changes in sinus diameters observed in our histological studies were due to the activation of Browicz–Kupffer cells. Because of their location and shape, these cells can interact with blood flow. Activation of Browicz–Kupffer cells is probably responsible for the more frequent occurrence of pit cells in liver sinuses [55,57].

Ito cells are “quiescent” in the normal liver, having received no stimuli to transform into a myofibroblastic state [56]. In our study, treatment with DON and DON + ZEN for 1, 3, or 6 weeks resulted in the presence of Ito cells with prominent cisterns of rough ER. The changes in Ito cells were probably related to their activation and transformation. They correlated with the occurrence of collagen fibers in the spaces of Disse.

The obtained data show that the administration of low doses of DON and ZEN resulted in prominent changes in the ultrastructure and histology of pig liver. In view of our results, even low levels of these mycotoxins, being below or close to No Observed Adverse Effect Level (NOAEL) values, should be considered as affecting the pig liver. The published data on the effects of such doses of DON and ZEN on the liver biochemistry are limited; however, they showed rather weak effects of these toxins on the serum activity of hepatic enzymes [58,59,60]. These findings agree with our morphological data showing that the responses of hepatocytes to intoxication with ZEN and DON seem to be adaptive. However, the changes in ER caused by ZEN and DON can affect the response of hepatocytes to other stressors or toxins [7,51]. In our opinion, special attention should be paid to the influence of ZEN and DON of the perilobular connective tissue, Browicz–Kupffer cells and Ito cells, which may lead to chronic damage of the liver. These effects cannot be detected with routine laboratory diagnostic methods.

## 3. Conclusions

Our current data provided strong evidence that the administration of low doses of DON and ZEN affected the ultrastructure and histology of pig liver. The changes caused by mycotoxins varied depending on the toxin and duration of intoxication. Both mycotoxins induced prominent modifications in the hepatocyte ER. The hepatocytes of ZEN-treated animals were characterized by extremely well-developed smooth ER, which comprised a dense network of narrow tubules. In the hepatocytes of DON-treated pigs, both rough and smooth ER were formed by an abundance of very short, dilated cisterns and vesicles. The effect of DON was greater than that of ZEN, as the ultrastructure of hepatocytes in pigs treated with DON + ZEN did not differ from that in DON-treated pigs. The total HAI scores in pigs receiving DON and DON + ZEN were significantly higher than that in the control animals, mainly because of the greater degree of hepatocyte necrosis and focal infiltration of inflammatory cells. Treatment with DON significantly increased the thickness of the perilobular connective tissue. The focal accumulation of collagen fibers was also observed in the perivascular spaces around the sinusoids, as shown by electron microscopy. These changes pointed to fibrosis as a potential effect of DON intoxication. Browicz–Kupffer cells were more frequently found and formed more prominent processes in pigs receiving mycotoxins, especially DON, and this phenomenon could be responsible for the dilation of sinusoids. Pit cells were more frequently observed in pigs treated with DON and DON + ZEN than in control animals. Examinations of glycogen and deposits of iron show the minor effect of the examined mycotoxins on these features. Our data demonstrate that DON and ZEN act both on hepatocytes and on liver immune/connective tissue cells. The response of hepatocytes to low doses of these toxins seems to be adaptive, and it is not related to serious cell damage. The influence of toxins on immune processes in the liver requires special attention because it can be destructive to the liver. Further molecular and biochemical studies are necessary to clarify the mechanisms of ZEN and DON toxicity in the liver.

## 4. Material and Methods

### 4.1. Animals, Toxins, and Experimental Design

The study was performed on 36 clinically healthy gilts of mixed breed (White Polish Big x Polish White Earhanging), with body weights of 25 ± 2 kg at the beginning of the experiment. The animals were purchased from a farm where they received feed without detectable amounts of ZEN, DON, α-zearalenol, aflatoxin, and ochratoxin. The pigs were fed twice daily and had free access to water.

The animals were divided into three experimental groups (D, Z, and M; *n* = 9 in each group) and a control group (C; *n* = 9). Group D received DON at a dose of 12 μg/kg BW per day, group Z received ZEN at a dose of 40 μg/kg BW per day, and group M received a mixture of DON + ZEN (ZEN 40 μg/kg BW + DON 12 μg/kg BW per day). The mycotoxins were synthesized and standardized at the Department of Chemistry, Faculty of Wood Technology, Poznań University of Life Sciences, Poland. The mycotoxins were administered orally during morning feeding in water-soluble capsules containing oat bran as a vehicle. The gilts were weighed every week to establish the amount of DON and ZEN administered to each animal. The animals in group C received capsules without mycotoxins.

The animals from the control and experimental groups were killed by intravenous administration of sodium pentobarbital (Vetbutal, Biowet, Poland) at a dose of 140–150 mg/kg and by exsanguination after 1, 3, and 6 weeks of experiment. Tissue samples were taken no longer than 3 min after cardiac arrest.

All procedures were carried out in compliance with Polish legal regulations for the determination of the terms and methods for performing experiments on animals and with the European Community Directive for the ethical use of experimental animals. The protocol was approved by the Local Ethical Council in Olsztyn (opinion No. 88/N of 16 December, 2009).

### 4.2. Histological Examination

Tissue samples (approximately 1 × 0.5 cm) were cut from the middle part of the liver. They were fixed in 4% paraformaldehyde in 0.1 M phosphate buffer (pH 7.4) for 48 h, dehydrated in ethanol (TP 1020; Leica, Wetzlar, Germany), and embedded in paraffin (EG1150; Wetzlar, Leica). Next, 4-µm-thick sections were prepared using an HM 340E microtome (Microm, Lugo, Spain) and stained with hematoxylin and eosin (HE), Mallory’s trichrome, PAS, and PB to detect iron using an automated multistainer ST 5020 (Leica, Wetzlar, Germany). For histological evaluation, the sections were scanned using a Mirax Desk scanner (Carl Zeiss, Oberkochen, Germany). The slides were signed in a way that prevented the people involved in the microscopic analysis from knowing the kind and duration of the animal treatment.

We evaluated the architectural changes in the liver, noting the organization of hepatic cords, the appearance of perilobular connective tissue, and the penetration of collagen into the parenchyma on Mallory- and HE-stained specimens.

Histological changes in the liver parenchyma were analyzed on HE-stained slides. The following parameters were evaluated:

- Ishak modified histology activity index (HAI) based on Ishak et al. [61] and modified by Stanek et al. [38]; parameters taken in consideration include portal, periportal, and acinar inflammation, focal or confluent necrosis, and hemorrhages;
- The presence of lymphoid follicles, steatosis, hepatocellular dysplasia, karyomegaly;
- The dilatation of hepatic sinusoids using the semi-counted method.

For morphometrical evaluations, the following parameters were determined:

- The cross-sectional area of lobules;
- The thickness of the interlobular connective tissue septa.

Measurements were performed on HE-stained sections using the Pannoramic Viewer 1.15 software (3D-Histech, Budapest, Hungary).

Glycogen content and iron accumulation in hepatocytes was evaluated in PAS- and PB-stained sections, respectively.

### 4.3. Ultrastructural Examination

Samples of liver tissue were collected from sites adjacent to the sampling sites for histological examination, and then immersion-fixed in a mixture of 1% paraformaldehyde and 2.5% glutaraldehyde in 0.2 M phosphate buffer (pH 7.4) for 2 h at 4 °C. Next, they were washed and post-fixed in 2% OsO_4_ in 0.2 M phosphate buffer (pH 7.4) for 2 h. After dehydration, the samples were embedded in Epon 812. Semi-thin sections were cut from each block of tissue, stained with 1% toluidine blue, and examined under a light microscope to choose the sites for preparing ultrathin sections. Ultrathin sections were cut using a Leica Ultracut III ultramicrotome (Leica, Wetzlar, Germany) and contrasted with uranyl acetate and lead citrate. They were examined with a Tecnai 12 Spirit G2 BioTwin transmission electron microscope (FEI, Hillsboro, OR, USA) equipped with two digital cameras: Veleta (Olympus, Tokyo, Japan) and Eage 4k (FEI, Hillsboro, OR, USA).

### 4.4. Statistical Analysis

The data from morphometric investigations were analyzed using one-way ANOVA with a Duncan test as a post-hoc procedure, and the data from semiquantitative analyses were analyzed using Kruskal–Wallis non-parametric ANOVA. Statistical analyses were performed using Statistica 10.0 software (StatSoft Polska, Cracow, Poland).

## Figures and Tables

**Table 1:** Semiquantitative analysis of fibrosis.

Duration of Mycotoxin Treatment	Group				Testing Results *p* < 0.05
	Control (C)	ZEN (Z)	DON (D)	DON + ZEN (M)	
1 week	0	0	1.0	2.67	C, Z < D; D < M
3 weeks	0	0	1.0	2.3	C, Z < D; D < M
6 weeks	0	0	1.33	3.0	C, Z < D; D < M

Description of the score used: 0, no fibrosis, perilobular tissue septa with small number of collagen, characteristic for the pig liver; 1, mild enlargement of portal areas and perilobular tissue septa; 2, large enlargement of portal areas and perilobular tissue septa, limiting plate fibrosis; 3, partial cirrhosis of some lobules. The values presented are means.

**Table 2:** Semiquantitative analysis of sinusoidal dilatation.

Duration of Mycotoxin Treatment	Group				Testing Results *p* < 0.05
	Control (C)	ZEN (Z)	DON (D)	DON + ZEN (M)	
1 week	0	3.00	3.67	3.33	C < Z, D, M
3 weeks	0.66	2.33	3.33	3.67	C < Z, D, M; Z < M
6 weeks	0.33	2.0	2.33	3.33	C < Z, D, M; Z, D < M

Description of the score used in estimating changes: 0, absence; 1, sporadic presence; 2, few presence; 3, middle presence; and 4, numerous presence.

**Table 3:** Semiquantitative analysis of glycogen content in the central and peripheral parts of liver lobules.

Time of Mycotoxin Treatment	Groups				Testing Results
	Control (C)	ZEN (Z)	DON (D)	DON + ZEN (M)	
Central part					
1 week	0	3.00	2.33	2.33	C < Z, D, M
3 weeks	2.66	3.33	2.33	3.00	−
6 weeks	3.00	2.66	3.00	3.33	−
Peripheral part					
1 week	0	4.00	4.00	3.33	C < Z, D, M
3 weeks	3.66	4.00	4.00	4.00	-
6 weeks	4.00	3.66	4.00	444	-

For a description of the score, see Table 2.

**Table 4:** Semiquantitative analysis of iron deposits.

Time of Mycotoxin Treatment	Groups				Testing Results
	Control (C)	ZEN (Z)	DON (D)	DON + ZEN (M)	
One week	1.00	1.00	1.00	1.00	-
Three weeks	1.00	1.66	1.00	2.00	C, D < Z, M
Six weeks	1.33	3.33	1.33	3.33	C, D < Z, M

For a description of the score, see Table 2.

## Supplementary Materials

The following are available online at https://www.mdpi.com/2072-6651/12/7/463/s1. Figure S1. Microphotographs of the histopathological parameters taken into account in HAI A. Portal inflammation. B. Periportal inflammation C. Acinar inflammation. D. Confluent necrosis. E. Focal lytic necrosis F. Hemorrhage, Figure S2. Dilatation of hepatic sinusoids in zone III (periportal) of acinus in a pig receiving DON + ZEN for 3 weeks. HE staining.

## References

[1] Cavret C., Lecoeur S. (2006). Fusariotoxin transfer in animals. Food Chem. Toxicol..

[2] Buszewska-Forajta M. (2020). Mycotoxins, invisible danger of feedstuff with toxic effect on animals. Toxicon.

[3] Nayakwadi S., Ramu R., Kumar Sharma A., Kumar Gupta V., Rajukumar K., Kumar V., Shirahatti P.S., Rashmi L., Basalingappa K.M. (2020). Toxicopathological studies on the effects of T-2 mycotoxin and their interaction in juvenile goats. PLoS ONE.

[4] Koselski M., Dziubińska H., Trębacz K., Sieprawska A., Filek M. (2019). The Role of SV Ion Channels under the Stress of Mycotoxins Induced in Wheat Cells—Protective Action of Selenium Ions. J. Plant Growth Regul..

[5] Sun Y., Wen J., Chen R., Deng Y. (2019). Variable protein homeostasis in housekeeping and non-housekeeping pathways under mycotoxins stress. Sci. Rep..

[6] Hallsworth J.E. (2018). Stress-free microbes lack vitality. Fungal Biol..

[7] Tiemann U., Brüssow K.P., Dannenberger D., Jonas L., Pöhland R., Jäger K., Dänicke S., Hagemann E. (2008). The effect of feeding a diet naturally contaminated with deoxynivalenol (DON) and zearalenone (ZON) on the spleen and liver of sow and fetus from day 35 to 70 of gestation. Toxicol. Lett..

[8] Jiang S.Z., Yang Z.B., Gao J., Liu F.X., Broomhead J., Chi F. (2011). Effects of purified zearalenone on growth performance, organ size, serum metabolities, and oxidative stress in postweaning gilts. J. Anim. Sci..

[9] Conková E., Laciaková A., Kovác A., Seidel H. (2003). Fusorial toxins and their role in animal disease. Vet. J..

[10] Alm H., Brussow K.P., Vanselow J., Tomek W., Danicke S., Tiemann U. (2006). Influence of Fusarium-toxins contaminated feed on initial quality and meiotic competence of gilt oocytes. Reprod. Toxicol..

[11] Prelusky D.B., Hartin K.E., Trenholm H.L., Miller J.D. (1988). Pharmacokinetic fate of 14C-labeled deoxynivalenol in swine. Fundam. Appl. Toxicol..

[12] Dänicke S., Valenta S., Döll S. (2004). On the toxicokinetics and the metabolism of deoxynivalenol (DON) in the pig. Arch. Anim. Nutr..

[13] Dänicke S., Swiech E., Buraczewska L., Ueberschar K.H. (2005). Kinetics and metabolism of zearalenone in young female pigs. J. Anim. Physiol. Anim. Nutr..

[14] Pinton P., Oswald I.P. (2014). Effects of deoxynivalenol and other Type B trichothecenes on the intestine: A review. Toxins.

[15] Gerez J.R., Pinton P., Callu P., Grosjean F., Oswald I.P., Bracarense A.P. (2015). Deoxynivalenol alone or in combination with nivalenol and zearalenone induce systemic histological changes in pigs. Exp. Tox. Pathol..

[16] Lewczuk B., Przybylska-Gornowicz B., Gajęcka M., Targońska K., Ziółkowska N., Prusik M., Gajęcki M. (2016). Histological structure of duodenum in gilts receiving low doses of zearalenone and deoxynivalenol in feed. Exp. Toxicol. Pathol..

[17] Przybylska-Gornowicz B., Tarasiuk M., Lewczuk B., Prusik M., Ziółkowska N., Zielonka Ł., Gajęcki M., Gajęcka M. (2015). The effects of low doses of two Fusarium toxins, zearalenone and deoxynivalenol, on the pig jejunum. A light and electron microscopic study. Toxins.

[18] Przybylska-Gornowicz B., Lewczuk B., Prusik M., Hanuszewska M., Petrusewicz-Kosińska M., Gajęcka M., Zielonka Ł., Gajęcki M. (2018). The effects of Deoxynivalenol and Zearalenone on the pig large intestine. A light and electron microscopy study. Toxins.

[19] Doll S., Danicke S. (2011). The fusarium toxins deoxynivalenol (DON) and zearalenone (ZON) in animal feeding. Prev. Vet. Med..

[20] Gajęcki M. (2002). Zearalenone—Ndesirable substances in feed. Pol. J. Vet. Sci..

[21] Malekinejad H., Maas-Bakker R.F., Fink-Gremmels J. (2005). Bioactivation of zearalenone by porcine hepatic biotransformation. Vet. Res..

[22] Olsen M., Kiessling K.H. (1983). Species differences in zearalenone-reducing activity in subcellular fractions of liver from female domestic animals. Acta Pharmacol. Toxicol..

[23] Miles C.O., Alistair L.W., Wilkins A.L., Neal R.T., Barry L.S., Ian G., Bryan G.S., Richard H.P. (1996). Ovine metabolism of zearalenone to alpha-zearalenol (Zeranol). J. Agric. Food Chem..

[24] Malekinejad H., Maas-Bakker R.F., Fink-Gremmels J. (2006). Species differences in the hepatic biotransformation of zearalenone. Vet. J..

[25] Dong M., Tulayakul P., LI J.Y., Manabe N., Kumagai S. (2010). Metabolic conversion of zearalenone to alpha-zearalenol by goat tissues. J. Vet. Med. Sci..

[26] Yang C., Song G., Lim W. (2020). Effects of endocrine disrupting chemicals in pigs. Environ. Pollut..

[27] Wan L.Y.M., Turner P.C., El-Nezami H. (2013). Individual and combined cytotoxic effects of Fusarium toxins (deoxynivalenol, nivalenol, zearalenone and fumonisins B1) on swine jejunal epithelium cells. Food Chem. Tox..

[28] Ji J., Cui F., Pi F., Zhang Y., Li Y., Wang J., Sun X. (2017). The antagonistic effect of mycotoxins deoxynivalenol and zearalenone on metabolic profiling in serum and liver mice. Toxins.

[29] Van Le Thanh B., Lemay M., Bastien A., Lapointe J., Lessard M., Chorfi Y., Guay F. (2016). The potential effects of antioxidant feed additives in mitigating the adverse effects of corn naturally contaminated with fusarium mycotoxins on antioxidant systems in the intestinal mucosa, plasma, and liver in weaned pigs. Mycotoxin Res..

[30] Alassane-Kpembi I., Kolf-Clauw M., Gauthier T., Abrami R., Abiola F.A., Oswald P., Puel O. (2013). New insights into mycotoxin mixtures: The toxicity of low doses of Type B trichothecenes on intestinal epithelial cells is synergistic. Toxicol. Appl. Pharmacol..

[31] Reddy K.E., Jeong J., Lee Y., Lee H.-J., Kim M.S., Kim D.W., Jung H.-J., Choe C., Oh Y.K., Lee S.D. (2018). Deoxynivalenol- and zearalnone-contaminated feeds alter gene expression profiles in the livers of piglets. Asian-Australas. J. Anim. Sci..

[32] Zollner P., Jodlauber J., Kleinova M., Kahlbacher H., Kuhn T., Hochsteiner W., Lindner W. (2002). Concentrations levels of zearalenone and its metabolities in urine, muscle tissue, and liver samples of pigs fed with mycotoxins-contaminated oats. J. Agric. Food Chem..

[33] Mikami O., Yamaguchi H., Murata H., Nakajima Y., Miyazaki S. (2010). Induction of apoptotic lesions in liver and lymphoid tissues and modulation of cytokine mRNA expression by acute exposure to deoxynivalenol in piglets. J. Vet. Sci..

[34] Renner L., Kahlert S., Tesch T., Bannert E., Frahm J., Barta-Böszörményi A., Kluess J., Kersten S., Schönfeld P., Rothkötter H.J. (2017). Chronic DON exposure and acute LPS challenge: Effects on porcine liver morphology and function. Mycotoxin Res..

[35] Povero D., Busletta C., Novo E., di Bonzo L.V., Cannito S., Paternostro C., Parola M. (2010). Liver fibrosis: A dynamic and potentially reversible process. Histol. Histopathol..

[36] Greaves P. (2012). Histopathology of Preclinical Toxicity Studies: Interpretation and Relevance in Drug Safety Evaluation.

[37] Weber S.N., Wasmuth H.E. (2010). Liver fibrosis: From animal models to mapping of human risk variants. Best Pract. Res. Clin. Gastrooenterol..

[38] Stanek C., Reinhardt N., Diesing A.-K., Nossol C., Kahlert S., Panther P., Kluess J., Rothkotter H.-J., Kuester D., Brosig B. (2012). A chronic oral exposure of pigs with deoxynivalenol partially prevents the acute effects of lipopolisaccharides on hepatic histopathology and blood clinical chemistry. Toxicol. Res..

[39] Friedman S.L., McQuaid K.R., Grendell J.H. (2003). Current Diagnosis & Treatment in Gastroenterology.

[40] Mills S.E. (2007). Histology for Pathologists.

[41] Foster J.R. (2005). Spontaneous and drug-induced hepatic pathology of the laboratory beagle dog, the cynomolgus macaque and the marmoset. Toxicol. Pathol..

[42] Van Amersfoort E.S., Van Berkel T.J.C., Kuiper J. (2003). Receptors, Mediators, and Mechanisms Involved in Bacterial Sepsis and Septic Shock. Clin. Microbiol. Rev..

[43] Coulombe R.A. (2003). Pyrrolizidine alkaloids in foods. Adv. Food Nutr. Res..

[44] Winkler K., Poulsen H. (1975). Liver disease with periportal sinusoidal dilatation. A possible complication to contraceptive steroids. Scand. J. Gastroenterol..

[45] Ribero D., Wang H., Donadon M., Zorzi D., Thomas M.B., Eng C., Chang D.Z., Curley S.A., Abdalla E.K., Ellis L.M. (2007). Bevacizumab improves pathologic response and protects against hepatic injury in patients treated with oxaliplatin-based chemotherapy for colorectal liver metastases. Cancer.

[46] Halliday J.W., Searle J. (1996). Hepatic iron deposition in human disease and animal models. Biometals.

[47] Graham R.M., Chua A.C., Herbison C.E., Olynyk J.K., Trinder D. (2007). Liver iron transport. World J. Gastroenterol..

[48] Deugnier Y., Turlin B. (2007). Pathology of hepatic iron overload. World J. Gastroenterol..

[49] Pietrangelo A. (2016). Mechanisms of iron hepatotoxicity. J. Hepatol..

[50] Yiannikourides A., Latunde-Dada G.O. (2019). A Short Review of Iron Metabolism and Pathophysiology of Iron Disorders. Medicines.

[51] Cheville N.F. (2009). Ultrastructural pathology. The Comparative Cellular Basis of Diseases.

[52] Hutterer F., Schaffner F., Klion F.M., Popper H. (1968). Hypertrophic, Hypoactive Smooth Endoplasmic Reticulum: A Sensitive Indicator of Hepatotoxicity Exemplified by Dieldrin. Science.

[53] Burton G.J., Yung H.W. (2011). Endoplasmic reticulum stress in the pathogenesis of early-onset pre-eclampsia. Pregnancy Hypertens..

[54] Han C.Y., Rho H.S., Kim A., Kim T.H., Jang K., Jun D.W., Kim J.W., Kim B., Kim S.G. (2018). FXR inhibits endoplasmic reticulum stress-induced NLRP3 inflammasome in hepatocytes and ameliorates liver injury. Cell Rep..

[55] Crawford J.M., Bioulac-Sage P., Hytiroglou P., Burt A.D., Ferrell L.D., Hübscher S.G. (2018). Structure, Function, and Responses to Injury. Macsween’s Pathology of the Liver.

[56] Hasegawa D., Wallace M.C., Friedman S.C., Gandhi C.R., Pinzani M. (2015). Chapter 4—Stellate Cells and Hepatic Fibrosis. Stellate Cells in Health and Disease.

[57] Peng H., Wisse E., Tian Z. (2016). Liver natural killer cells: Subsets and roles in liver immunity. Cell Mol. Immunol..

[58] Gajęcka M., Tarasiuk M., Zielonka Ł., Dąbrowski M., Gajęcki M. (2016). Risk assessment for changes in the metabolic profile and body weights of pre-pubertal gilts during long-term monotonic exposure to low doses of zearalenone (ZEN). Res. Vet. Sci..

[59] Nicpoń J., Sławuta P., Nicpoń J. (2016). Effect of zearalenone toxicosis on the complete blood cell count and serum biochemical analysis in wild boars. Vet. Med..

[60] Holanda D.M., Kim S.W. (2020). Efficacy of Mycotoxin Detoxifiers on Health and Growth of Newly-Weaned Pigs under Chronic Dietary Challenge of Deoxynivalenol. Toxins.

[61] Ishak K., Baptista A., Bianchi L., Callea F., De Groote J., Gudat F., Denk H., Desmet V., Korb G., MacSween R.N.M. (1995). Histological grading and staging of chronic hepatitis. J. Hepatol..

