# Supplementary material for: Effects of Deoxynivalenol and Zearalenone on the Histology and Ultrastructure of Pig Liver
Source: Toxins (Basel). 2020 Jul 20;12(7):463. doi: 10.3390/toxins12070463 (PMC7404993; doi:10.3390/toxins12070463)
Supplement: Supplementary file 1 [file toxins-12-00463-s001.pdf]

Supplementary Materials: Effects of Deoxynivalenol and Zearalenone on the Histology and Ultrastructure of Pig Liver

Natalia Skiepko, Barbara Przybylska-Gornowicz, Magdalena Gajęcka, Maciej Gajęcki and Bogdan Lewczuk

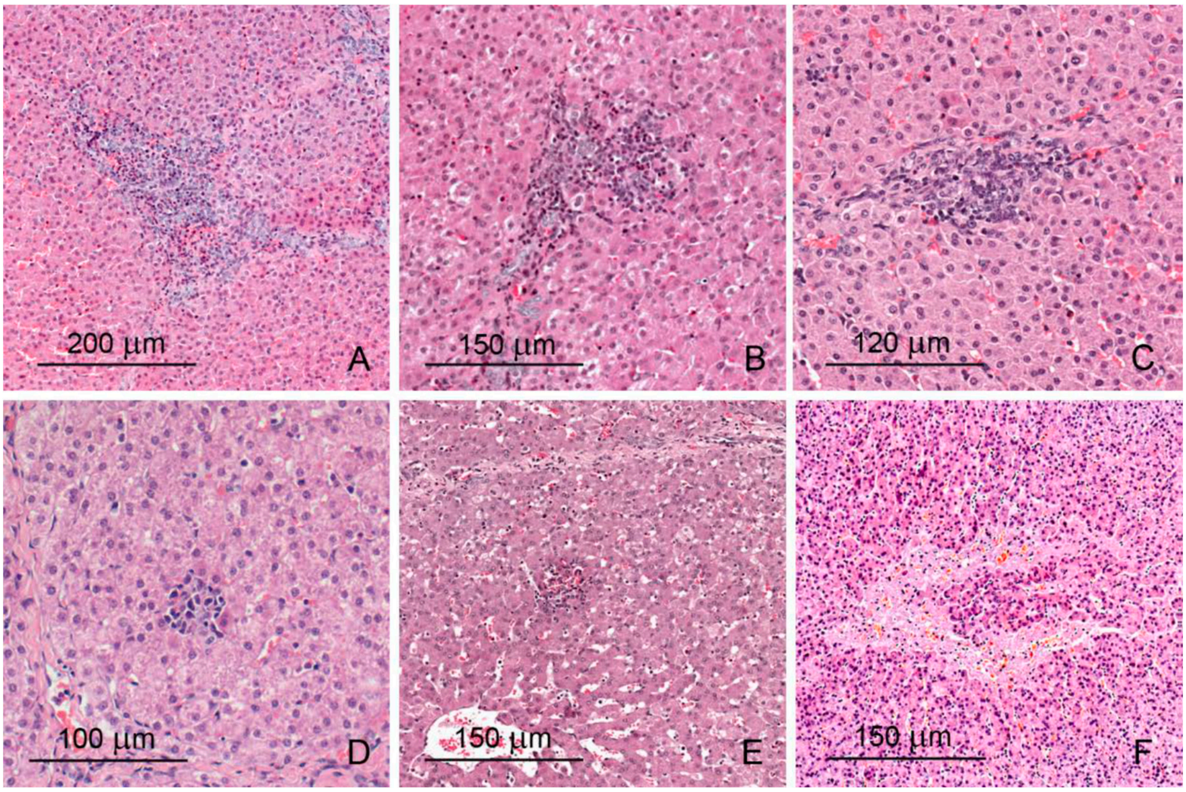

Figure S1. Microphotographs of the histopathological parameters taken into account in HAI. **A**. Portal inflammation. **B**. Periportal inflammation **C**. Acinar inflammation. **D**. Confluent necrosis. **E**. Focal lytic necrosis **F**. Hemorrhage.

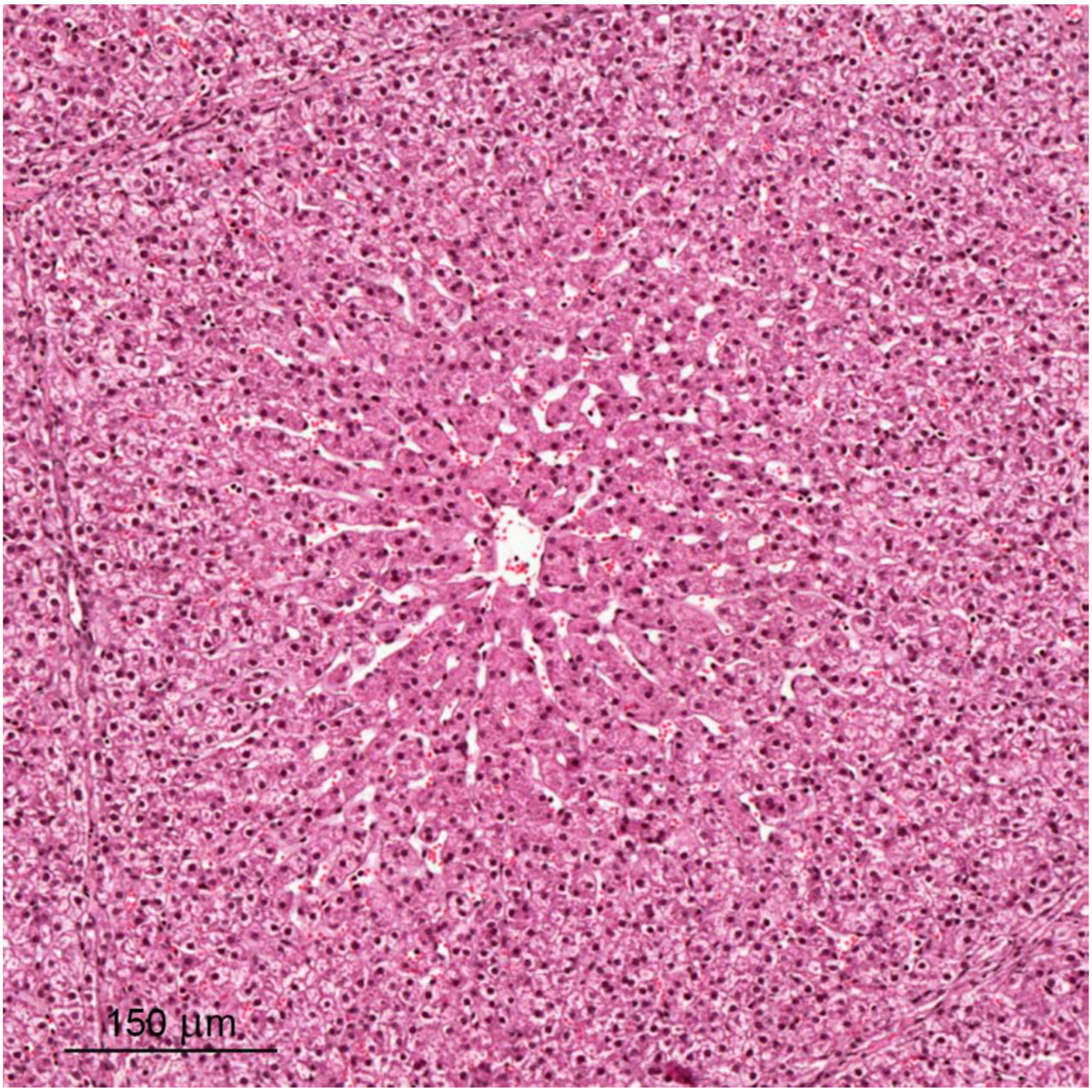

Figure S2. Dilatation of hepatic sinusoids in Zone III (periportal) of acinus in a pig receiving DON + ZEN for 3 weeks. HE staining.
